# Exploring the comparative adequacy of a unimanual and a bimanual stimulus-response setup for use with three-alternative choice response time tasks

**DOI:** 10.1371/journal.pone.0281377

**Published:** 2023-03-15

**Authors:** Anton Öttl, Jonathan D. Kim, Dawn M. Behne, Pascal Gygax, Jukka Hyönä, Ute Gabriel

**Affiliations:** 1 Department of Psychology, Norwegian University of Science and Technology, Trondheim, Norway; 2 Department of Psychology, University of Fribourg, Fribourg, Switzerland; 3 Division of Psychology, University of Turku, Turku, Finland; Federal University of Paraiba, BRAZIL

## Abstract

Research often conceptualises complex social factors as being distinct binary categories (e.g., female vs male, feminine vs masculine). While this can be appropriate, the addition of an ‘overlapping’ category (e.g., non-binary, gender neutral) can contextualise the ‘binary’, both for participants (allowing more complex conceptualisations of the categories than the ‘either/or’ conceptualisation in binary tasks), and for the results (by providing a neutral baseline for comparison). However, it is not clear what the best response setup for such a task would be. In this study, we explore this topic through comparing a unimanual (*N* = 34) and a bimanual response setup (*N* = 32) for use with a three-alternative choice response time task. Crucially, one of the stimulus categories (‘mixed’) was composed of stimulus elements from the other two stimulus categories used in that task (Complex Task). A reference button task was included to isolate the motoric component of response registration (Simple Task). The results of the simple task indicated lower motoric costs for the unimanual compared to the bimanual setup. However, when statistically controlling for these motoric costs in the complex task, the bimanual setup had a lower error rate and faster response times than the unimanual setup. Further, in the complex task error rates and response times were higher for the mixed than the matched stimuli, indicating that responding to mixed stimuli is more challenging for encoding and/or decision making processes. This difference was more pronounced in the unimanual than the bimanual setup. Taken together these results indicate that the unimanual setup is more adequate for the reference button task, whereas the intricacy of overlapping categories in the complex task is better contained in the bimanual setup, i.e. when some response alternatives are allocated to one hand and other alternatives to the other hand.

## 1. Introduction

Research into the cognitive activation of social categories predominantly uses tasks that are limited to binary responses (e.g. gender—“female” vs “male”, [1, 2]). In an applied context, this limits the potential generalisability of findings, as the question of whether an intervention is successful is only tested indirectly and without nuance; for example, an intervention examining whether gender-inclusive language removes gender bias would only be said to be successful if, when responding to the task, participants showed *no* difference in accuracy and speed when responding to both feminine and masculine items (e.g., [3]). A potential solution to this limitation is the use of a task with a third response alternative that represents an explicitly intermediatory category (e.g., gender—“both female and male”), but it is not clear how such a stimulus-response interface should best be designed. This study aims to help clarify this question. Its specific motivation was to evaluate response set-ups for the development of an experimental paradigm investigating gender information–more precisely, a paradigm that supplements the usual ‘male’ *or ‘*female’ categories with a third ‘male *and* female’ category. This puts certain constraints on the design alternatives explored, including that this study is limited to exploring tasks with three-alternative stimulus/response designs. However, as the issues raised are of a more general nature, and hence highly relevant for other domains, in the following section they will be discussed in a general manner to facilitate cross-domain translation.

Evaluation of response setups is based on the connection between stimuli and response options. In a stimulus-response experimental paradigm, the process under investigation is that of perceiving, processing, and responding to a presented stimulus (e.g., [4]). This includes encoding (i.e., processing of relevant sensory input), accumulation of evidence to reach a decision, and motoric response execution [5].

The current study evaluates response setups for use with Choice Response Time (CRT) tasks, where participants are instructed to sort varied stimuli into predetermined categories as quickly and accurately as possible [6]. CRT tasks measure both ‘Choice’ (i.e., how accurately participants respond to stimuli in each category) and Response Time (i.e., how fast they respond *accurately* to stimuli in each category; e.g., [7]), as information in line with our beliefs and expectations is responded to faster and more accurately than information against them (e.g., [8]). These tasks are often used to examine Stimulus-Response factors, as the requirement for fast identification and differential responses to stimuli based on its associated category enables explorations of both physiological and psychological factors related to Stimulus-Response behaviour [7].

The response setup a study uses impacts on both accuracy and response times, as specifics of the setup (and specific implementations of response alternatives) can affect encoding and decision processes, and/or the time taken for the motoric execution of the response (e.g., [5]). For example, in an experiment examining colour discrimination ability, participants might be instructed to press ‘e’ if they see a blue dot, ‘y’ if they see a red dot, and ‘i’ if they see a yellow dot. If participants are instructed to rest their digit fingers on the ‘e’ and ‘i’ keys, they can press these buttons easily, but would be forced to move a finger to press the ‘y’ key. Such differences in the motoric effort required to register responses to different stimuli can be seen to affect responses and response times in a systematic (i.e., per response alternative) manner.

Further, the use of more than two response alternatives may come with a more complex internal logical structure than is observed in two-alternative studies. For example, blue, red, and yellow dots are all stimuli in primary colours, so they may be interpreted as separate (blue ≠ red ≠ yellow), but blue, green, and yellow dots may instead be interpreted as a continuum that connects two primary colours through their secondary colour (blue ⇔ green ⇔ yellow). While response setups can be designed to cater to either of these situations, researchers must deliberately decide on whether, and how, (implicit and explicit) relationships between the stimuli will be reflected in the response alternatives, as this will eventually add extraneous variability to responses and response times.

As overlapping stimulus categories require at least two other stimulus categories, they can only appear in three+ alternative designs, making their use a far less common occurrence. As such, that they have received little attention is perhaps unsurprising. However, exploring the impact of overlapping stimulus categories is essential for ensuring that experiments use the response setups with the minimal possible impact on the results. This study is therefore aimed at investigating the intersection between stimulus categories (individual vs. overlapping) and conceptualisations (one- vs. two-dimensional) of response categories. More specifically, we discuss and evaluate two specific response setups for a CRT task with one overlapping category.

### 1.1. Background

Stimulus-Response (S-R) compatibility can be defined as the level to which an individual’s perception of a given stimulus is compatible with the action they need to undertake when the stimulus is presented (e.g., [9, 10]). Under the Salient-Feature Coding Hypothesis [11–14] S-R compatibility is high when salient features of both stimuli and response options correspond, even when they do not directly overlap. The higher the level of S-R compatibility, the lower the level of attentional control (and other cognitive factors) required from participants to respond accurately [15]. Stimulus information is therefore translated into accurate physical responses more quickly for setups with high S-R compatibility [9]. Further, when participants are instructed to respond *quickly*, low S-R compatibility can result in heightened error rates, as participants respond before they have finished interpreting the stimuli (e.g., [9, 15]). The S-R compatibility for experimental setups can therefore be determined by examining response times and error rates, with the two measures usually being positively correlated for compatibility manipulations ([16] p. 11).

S-R compatibility is composed of two important aspects; set-level compatibility and element-level compatibility [10, 17–19]. Set-level compatibility refers to the specific properties of the stimulus and response sets and their interaction [10, 18]. As an example, Fitts and Seeger (1953) [10] found that set-level compatibility is highest when the spatial arrangement of stimuli is most closely matched by the spatial definition of responses. For example, if four spatially arranged stimuli are positioned in a square, spatially defined responses that mimic a square will have higher set-level compatibility than spatially defined responses that mimic a diamond. Element-level compatibility refers to the properties of individual elements within stimulus and response sets [17, 18]. As an example, Fitts and Deininger (1954) [17] found that, when spatial arrangement of stimuli and spatial definition of responses is matched (e.g., in relation to previous example, both are ‘square’ shaped), element-level compatibility differs based on the connection between the specific spatial position of a stimuli in relation to how it should be responded to. Specifically, they found that a given S-R setup had the highest element-level compatibility if the buttons used to respond matched the locations of the stimulus on the screen, less compatibility if the buttons were ‘mirrors’ of the location of the stimulus on the screen, and the least compatibility if the buttons were randomly assigned as to which stimulus they correspond to. For example, for a bimanual two-alternative task, responding to a stimulus presented on the left of the screen with the hand on the left is highly compatible, while responding to a stimulus presented on the left of the screen with the hand on the right is not very compatible [14, 18].

The examples used to illustrate the difference between set- and element-level compatibility are based on Spatial-Anatomical Relationships, as it is relevant for this study. However, there is a broad range of other factors not examined herein that affect S-R compatibility (see [4] for an overview), including Population Stereotypes (culturally-specific associations between stimuli and response options, e.g., [20]), and Stimulus-Response Intensity (relationship between the force required to register a response and the intensity of the stimuli, e.g., [21]).

This study investigates spatial-anatomical relationships’ influence on set-level S-R compatibility–in particular, on the correspondence between stimulus and response set structures for tasks that include an ‘overlapping’ stimulus/response category. Previous research into set-level S-R compatibility has often utilised more simple CRT tasks where stimuli (“A”, “B”, “C”, etc.) have a high element-level S-R compatibility (e.g., [10, 22]), including modalities under which the stimuli (audio/text/spatial) and/or responses (spoken/written/manual) that were presented differed within or across experiments (e.g., [23, 24]). Further, research (e.g., [25–27]) has examined task-switching paradigms where stimuli activate multiple potential response options simultaneously (e.g., “A&1”, “B&2”, “C&3”, with participants instructed to respond based on only one dimension (e.g., either numeric [1, 2, or 3] or alphabetical [A, B, or C]). Less common has been examinations of more complex CRT tasks where stimulus categories may *conceptually* overlap with each other, while individually only corresponding to single response options. This is important as research (e.g., [15]) has found that participants may conceptualise three+ stimuli categories either individualistically (A [not B or C] vs. B [not A or C] vs. C [not A or B]) or as a continuum (A ⇔ B ⇔ C, e.g., child ⇔ adult ⇔ elder). This is especially the case when purposefully ‘overlapping’ stimulus categories are used; a factor which, to our knowledge, has not been previously examined outside of task-switching paradigms.

In this study, overlapping stimulus categories are defined as stimulus categories that are composed of stimulus elements that are utilised independently in isolation in *multiple* other stimulus categories. Extending the above example, if B (e.g., one female and one male face) is a stimulus category overlapping with A (e.g., two female faces) and C (e.g., two male faces), then B would contain stimulus elements from both A and C, but A and C would not directly share any stimulus elements. When overlapping stimulus categories are utilised in an experiment such that the independent stimulus categories (A and C) share stimulus elements with the overlapping stimulus categories (B), this leads to the perception of the stimulus categories together forming some variety of continuum (and with B taking on the label of ‘A&C’), although the conceptualisation of this continuum can differ greatly. Further extending the example, the stimulus categories can be conceptualised either in the form ‘A only ⇔ A&C ⇔ C only’, or in the form ‘A [but not C], C [but not A], both A and C’. The difference therefore is of dimensions; the former (one-dimensional) suggests a one (stimulus presented) by three (A vs. A&C vs. C) design, with participants selecting the one stimulus category that matches what they see (two female faces, two male faces, or one female and one male face), while the second (two-dimensional) suggests a two (stimulus presented vs. stimulus not presented) by two (A vs. C) design, with participants identifying if they specifically do *or do not* see stimulus elements from the different stimulus categories (is a female face seen [yes/no], is a male face seen [yes/no]). These approaches come with different interpretations of the *nature* of overlapping stimulus categories. In the one-dimensional approach, an overlapping stimulus category is concrete and unique (A&C ≠ A; A&C ≠ C; A ≠ C). In the two-dimensional approach, an overlapping stimulus category is the sum of its parts (A&C = A + C; A ≠ C), and therefore neither concrete nor unique. In consequence, overlapping stimulus categories are *explicitly* represented in the one-dimensional approach, but only *implicitly* represented in the two-dimensional approach.

Conceptualisation of a stimulus set as being one-dimensional should result in a higher set-level SR compatibility with a unimanual response setup, as unimanual responses reflect a gradual change from exclusively A to exclusively C by using, for example, three consecutive fingers on one hand. However, unimanual responses may be inadequate for experiments where participants are more likely to conceptualise a stimulus set of being two-dimensional due to the conceptual disconnect between stimulus and response categories. As such, experimental tasks where the two-dimensional conceptualisation is more likely should result in a higher set-level SR compatibility with bimanual response options, where one finger (or hand) is associated with one stimulus category, another finger (or hand) is associated with a second stimulus category, and both fingers (or hands) are used to reply when stimulus in the third stimulus category (i.e., the overlapping category). Whether a unimanual or bimanual implementation is more adequate for a task may be an empirical question, as the participants´ experience and knowledge, and how those may impact their conceptualisation of, and performance during, the experiment is unknown a-priori. As such, the present study examines the comparative adequacy of one unimanual and one bimanual response setup for use with a three-alternative CRT task that contains stimulus categories that can be equally conceptualised as one- or two-dimensional.

For many studies motoric costs are not a barrier, as responses can be counterbalanced across conditions. However, when stimulus and/or response elements are shared across conditions, such as with overlapping stimulus categories, it is likely that counterbalancing cannot occur. In such cases, one must consider the motoric factors associated with a given setup, as they may be directly confounded with the perceptual or cognitive processes being investigated (e.g., [28–30]). To evaluate different response setups, one therefore needs to find a way to isolate the motoric component of response registration from the measured response and response time to ensure that variation in choices and response times is not a confound of variation in difficulties in motoric execution. To this, the present study included a reference button task that allows for an evaluation of the response setups with reference to motoric execution of the response and can be used to measure (and statistically control) baseline motoric costs for different response setups (e.g., [30, 31]).

**Fig 1:**
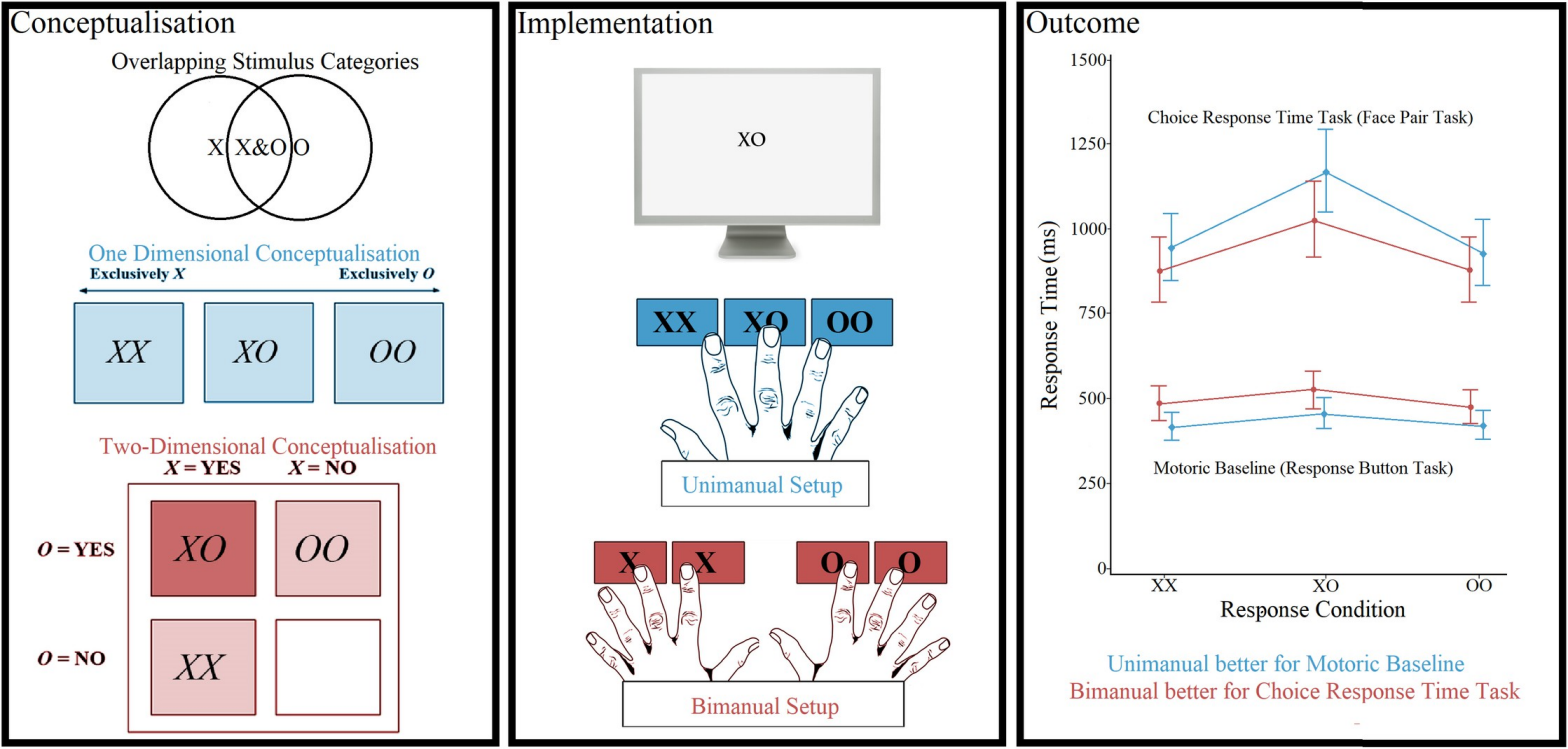
Visual abstract.

In summary, response setups for use with three+ alternative choice designs can offer competing conceptualisations that stimulus-response setups can draw from, especially as paradigm complexity increases. As such, research is needed to determine the best stimulus-response setup for use with three+ alternative choice designs of varying complexities. This study explores this topic, through comparing manual response setups (one unimanual, one bimanual) for use with visual stimuli in a three-alternative CRT task with overlapping stimulus categories. A summary of the conceptualisation, implementation, and outcome of this study can be seen in Fig 1.

### 1.2. The present study

The data for this study was collected with a unimanual and a bimanual response setup in a reference button task and a three-alternative choice response task with overlapping stimulus categories. The reference button task was included to evaluate differences in motoric execution of the respective response options, allowing for motoric effects to be distinguished from conceptual differences in the stimulus-response mapping (one-dimensional vs. two-dimensional conceptualisation). As the former task purely involves perception and response execution, while the latter task also involves evaluation of the presented stimuli, the former is referred to as “Simple Task” and the latter as “Complex Task”.

The data presented was recorded as part of a larger project examining gender representation. As a consequence, Response Setup was examined as a between-participant factor. During the study, participants’ responses, response times, and eye tracking data was gathered. Responses and response times were examined to allow for S-R compatibility to be examined per response setup and task. Eye tracking data was gathered as part of a gaze-contingent technique, where the presentation of stimuli was triggered when participants looked directly at a fixation cross presented on the screen. This data only revealed relevant information for the Complex Task, but did not add much to the overall picture. As such, eye tracking analysis is not discussed in the body of the article, but the results of the eye tracking analysis for the Complex Task can be found in S1 Appendix.

For the Simple Task, stimulus images were graphical representations of the response buttons with the respective button(s) being highlighted that participants needed to press to respond correctly. For the Complex Task, stimulus images were pairs of gendered faces. The overlapping stimulus category was one female and one male face, while the independent stimulus categories were composed of two faces of the same gender (i.e., two female or two male faces). Under the unimanual setup, responses were given through three buttons, with participants pressing one designated button to respond to each response condition. Under the bimanual setup, responses were given through four buttons, with participants pressing two designated buttons to respond to each response condition (two with either the left or right hand for the matched-response categories, or one with each hand for the mixed-response condition). We aim at comparatively evaluating the adequacy of the unimanual and bimanual setups for the Complex Task.

#### 1.2.1. Simple Task: Motoric difficulty in unimanual vs. bimanual response setups

As the stimuli used in this task in both setups closely match the actions participants are required to take to respond (equally high level of S-R compatibility), we assume that differences in responses and response times essentially reflect differences in the motoric execution of the response, and therefore assume that they can be used as an estimate of the motoric difficulty/costs associated with either setup. As such, the analysis for the Simple Task focused on the important motoric aspects associated with each response setup. Further, as this is such a simple task, we expect very small error rates for both setups. The core motoric factor for the unimanual setup is finger use. As the unimanual setup used three-buttons that are responded to with a single hand, the buttons can be seen to have been ‘assigned’ a finger; namely, the digit, middle, and ring finger of the participants’ dominant hand. In contrast to the index finger, the ring and middle fingers do not have independent flexors, making it more difficult to independently move these fingers [32]. Research based on simple response time tasks (i.e., finger responses following a visual signal that highlights the key[s] required for a correct response) suggests that reaction times are slower for the ring and middle fingers compared to the index and little fingers (e.g., [33] Exp.1; [34]). However, additional research varying the number of fingers included suggests that finger specific response times not only reflect motor factors associated with the specific finger, but also the identifiability/discrimination of stimulus and/or response, with an advantage for stimuli in the respective periphery of the visual field linked to the “outer fingers” (e.g. [33, 35]). Applied to our setup, we expect the results of the Simple Task to show that index finger responses are the fastest (finger motoric advantage and “outer finger” advantage), and that ring finger responses (“outer finger” advantage) will be faster than middle finger responses.

The core motoric factor for the bimanual setup is combined hand and finger use. The bimanual setup used four-buttons, with two buttons being assigned to the index and middle finger of the left hand, and the other two buttons being assigned to the index and middle finger of the right hand. Responses are either given unimanually (pressing two fingers from one hand) or bimanually (pressing the left and right index finger). For tasks requiring multiple response options, it is motorically easier for answers to be given unimanually compared to bimanually (e.g., [36]). In line with that, we expect longer response times for the synchronized bimanual responses than for the unimanual responses.

From a motoric perspective, the unimanual setup has overall comparatively small motoric costs, as participants do not need to coordinate two hands simultaneously [36]. We therefore expect *overall* faster responses for the unimanual setup than for the bimanual setup.

#### 1.2.2. Complex Task: Unimanual vs. bimanual setup

We assume that the motoric costs between the response options (left, middle, right) and between the set-ups (unimanual vs. bimanual) are in line with those in the simple task. However, we expect additional differences between the response options based on differences in the kind of decisions that participants need to make in the Complex Task. In this task (face pair task), participants are presented with two faces (face-pair) and have to decide whether they belong to people of the same gender (FF/MM) or to people of different genders (FM/MF). There is substantial evidence that participants in general are faster to decide that two stimuli are similar than to decide that they are different (see [37] for a review). One of the theoretical explanations for this effect is that the attributes displayed in one stimulus facilitate encoding/processing when they are displayed again in a second stimulus (e.g., [38] Exp. 4; [39]). In a similar vein, the face-pairs can be considered as two simultaneously presented stimuli, and therefore it can be assumed that responses to the mixed category (FM/MF) will be slower (and less accurate) than to the matched categories (FF and MM).

Furthermore, and most importantly, we ask whether the concrete example (female- and male-ness) is better conceptualised as a single dimension (as reflected in the unimanual setup) or two separate dimensions (as reflected in the bimanual setup). There has been discussion around whether ‘femaleness’ and ‘maleness’ should be looked upon as the ends of a single dimension, or as two separate dimensions (e.g., [40]). However, as our Complex Task (face pair task) relies on materials selected to reflect femaleness and maleness (as opposed to androgyny), we consider a two-dimensional structure to be a more adequate representation of the specific task at hand. More specifically, in the Complex Task we consider the bimanual four-button setup to have a comparatively higher level of S-R compatibility than the unimanual three-button setup for three reasons. First, the number of stimulus elements (two faces shown at the same time) is congruent with the number of response elements in the bimanual (two buttons pressed simultaneously) but not in the unimanual setup (one button pressed). Second, given the (arguably) dichotomous relationship between the female and the male response categories, compatibility is enhanced by linking the responses for the two matched-response categories (FF, MM) to different hands. Third, since the mixed-response condition constituted a combinatorial overlap between the two matched-response categories, the implementation of the mixed-response condition as a bimanual response forms a motoric analogue to the structure of the stimulus categories.

If the bimanual response set-up represents the Complex Task more adequately (higher stimulus-response compatibility) this would show in overall more accurate and faster responses in the bimanual compared to the unimanual setup. However, to justify the use of a complex response layout, any potential facilitation in the bimanual setup would at least need to compensate for the hypothesized increase in motoric difficulty as evaluated through the Simple Task (reference button task).

#### 1.2.3. Summary

To summarize, the bimanual response setup was designed to have a high level of S-R compatibility and correspondence for a CRT task with overlapping categories (face pair task) but, due to requiring the coordination of two hands simultaneously, increased motoric difficulty especially for the synchronized bimanual response. Conversely, the unimanual response setup was designed to have comparatively low level of correspondence and S-R compatibility for a complex three-alternative CRT task but, due to only requiring the finger coordination of one hand, reduced motoric difficulty.

The motoric effort associated with the response implementation will be assessed via the Simple Task (reference button task) with the following hypotheses:

- Overall, we expect a high level of correct responses for both response setups.
- For the unimanual setup, we expect faster responses for responses performed by the digit finger than for those performed by the middle or ring fingers, and faster responses by the ring finger than the middle finger. (H1)
- For the bimanual setup, we expect slower responses for synchronized bimanual responses than for the unimanual responses. (H2)
- We expect overall faster responses for the unimanual setup than for the bimanual setup. (H3)

With reference to the Complex Task (face pair task), our hypotheses are the following:

- We expect more accurate and faster responses to the matched categories (same-gender face pairs) than to the mixed category (different-gender face pairs). (H4)
- We expect more accurate and faster responses for the bimanual setup than for the unimanual setup when controlling for motoric costs, i.e. when participants’ error rate and response time from the Simple Task are included as a covariate. (H5)

## 2. Methods

**Fig 2:**
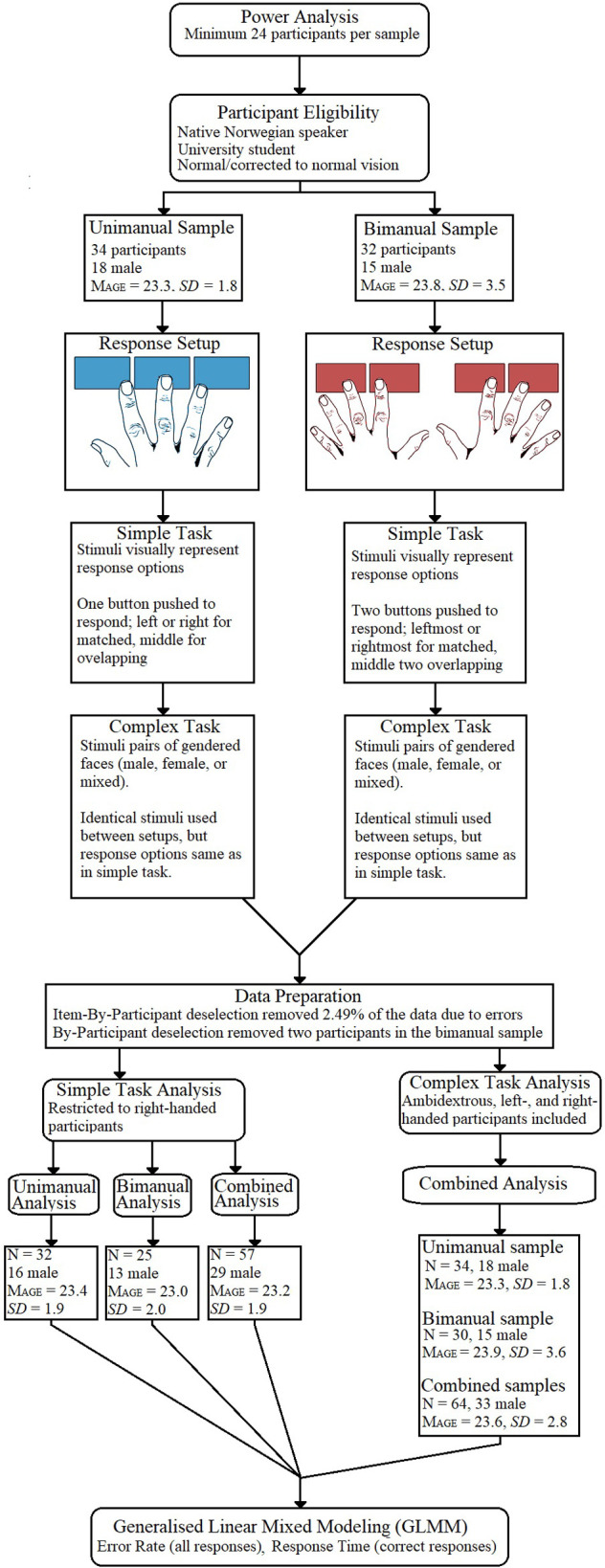
Flowchart presenting the experimental process.

A flowchart overview of the experimental process is provided in Fig 2. This covers sample selection, data collection, data preparation, and data analysis procedures for both unimanual and bimanual response setups.

### 2.1. Participants

A-priori power analysis indicated that, for *each* response setup, a sample size of n = 24 was required to test the interaction between stimulus and response categories with a medium effect size (alpha = .05, beta = .20, power = .80). Participants were eligible if they were native Norwegian speaking university-level students with normal or corrected to normal (i.e., glasses or contacts) vision, recruited at the Norwegian University of Science and Technology, Trondheim. Along with on-campus recruitment, aimed at selecting active students, these criteria were selected to ensure a healthy sample that was largely homogenous in terms of both age group and educational background. Participants were non-randomly assigned into either the unimanual or bimanual subsample.

For the unimanual subsample, 34 participants (18 male) with a mean age of 23.3 years (*SD* = 1.8) were recruited. For the bimanual subsample, 32 participants (15 male) with a mean age of 23.8 (*SD* = 3.5) were recruited. Since participants could take part regardless of hand dominance, both left- and right-handed participants were recruited into both subsamples..

### 2.2. Apparatus

The study was run on an air-gated Dell Latitude E5470 laptop with an Intel core i7-6820HQ CPU and 16gb RAM, running Windows 10 Education in 64-bit. Stimuli were displayed on a 22-inch computer display, with a resolution of 1680 x 1050 pixels, and a screen refresh rate of 60Hz. E-Prime software (v. 2.0.10.356 E-Prime Psychology Software Tools Inc., Pittsburgh, USA) was used for stimulus presentation and logging responses. Responses were collected using a Chronos response box that was positioned centrally to the participant and screen, and was connected to the presentation PC, while eye tracking information was collected using an SMI iView X RED500 system. To allow for the same response box to be used for all participants, stickers were used to label the buttons in line with the specifics of how each participant was required to respond. More specific information on these systems can be found in the project protocol (https://dataverse.no/dataset.xhtml?persistentId=doi:10.18710/WWPPCA#).

During the experiment the researcher sat facing the participant, with eye contact obstructed by the computer display between them. This allowed the participant as much privacy as possible, with the researcher able to indirectly monitor participants’ progress through access to both their gaze coordinates and a duplication of the stimulus screen on laptop screens facing away from the participant. Participants were instructed to rest their fingers (middle, index, and ring finger of the dominant hand for the unimanual setup; middle and index finger of both hands for the bimanual setup) on the response box.

### 2.3. Materials

To control for any order effect of stimulus position, the spatial arrangement (left—right) of the stimuli and responses was counterbalanced between participants, but kept constant across tasks. Consequently, stimuli were created in unique sets as described separately for each task.

#### 2.3.1. Simple task

**Fig 3:**
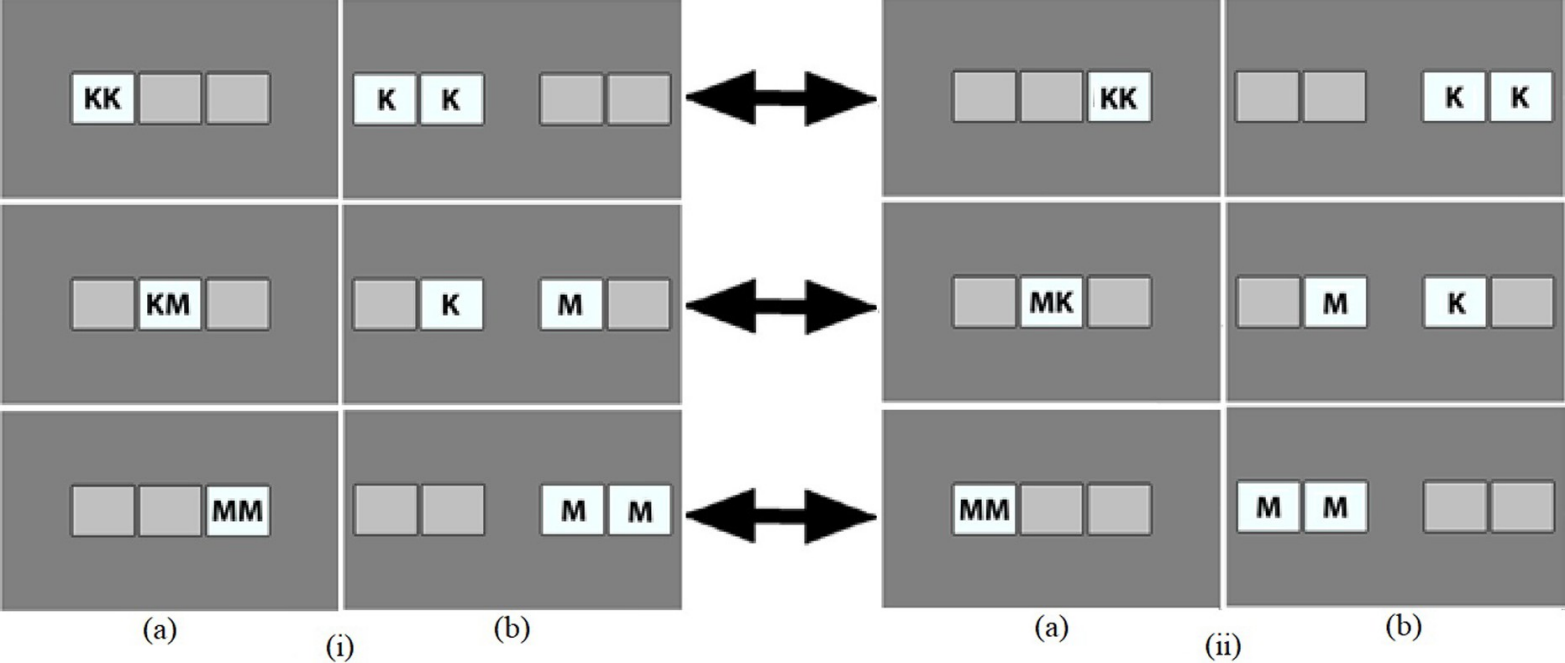
Stimuli used in the Simple Task in the unimanual (a) and bimanual setups (b). Note. Spatial arrangement: (i) KK left/MM right. (ii) MM left/KK right.

The sets of visual stimuli created for the simple task were basic visual representations of the button(s) participant needed to press to accurately respond to each trial (Fig 3). Unique stimuli were therefore created for each response setup, and were indicative of the spatial arrangement the participant was assigned to see (unimanual = Fig 3i[a], Fig 3ii[a]; bimanual = Fig 3i[b], Fig 3ii[b]). The stimuli were created in Adobe Photoshop CS4 Version 11.0, and were composed of grey boxes on a darker grey background. These boxes were visual representations of the buttons on the response box. The buttons that participants needed to press were highlighted by the corresponding boxes on screen being both labelled and a lighter shade of grey than the other boxes, as shown in Fig 3. This labelling was composed of one or two letters that matched the physical labels of the corresponding buttons on the response box. As this task also served as training for the Complex Task, the letters associated with the buttons were explicitly linked to gender. Since the study was conducted in Norwegian, the letters K and M were used, referring to *kvinnelige* [female] and *mannlige* [male], respectively. Aside from Fig 3, which displays the specific stimuli used, for ease of understanding F for *female* and M for *male* are used in the rest of the article. Further, as mixed stimuli could be in either the FM or MF order, these pairings are referred to solely as being ‘mixed’.

*2*.*3*.*1*.*1*. *Unimanual setup*. Two sets of three visual stimuli were created. In the first set (Fig 3i[a]), one stimulus displayed ‘FF’ in the left box, one stimulus displayed ‘MM’ in the right box, and the final stimulus displayed ‘FM’ in the centre box. In the second set (Fig 3ii[a]), the position of the letters of these stimuli were inverted from Fig 3i[a]. Set 1 was used when responses to the stimulus labelled ‘FF’ used the leftmost finger, while Set 2 was used when responses to the stimulus labelled ‘MM’ used the leftmost finger.

*2*.*3*.*1*.*2*. *Bimanual setup*. Two sets of three visual stimuli were created. In the first set (Fig 3i[b]), one stimulus displayed ‘F’ in the two leftmost boxes, another stimulus displayed ‘M’ in the rightmost two boxes, and the final stimulus displayed ‘F’ in the centre left box and ‘M’ in the centre right box. In the second set (Fig 3ii[b]), the position of the letters of these stimuli were inverted from Fig 3i[b]. Set 1 was used when responses to the stimulus labelled ‘FF’ used the left hand, while Set 2 was used when responses to the stimulus labelled ‘MM’ used the left hand.

#### 2.3.2. Complex task (both setups)

**Fig 4:**
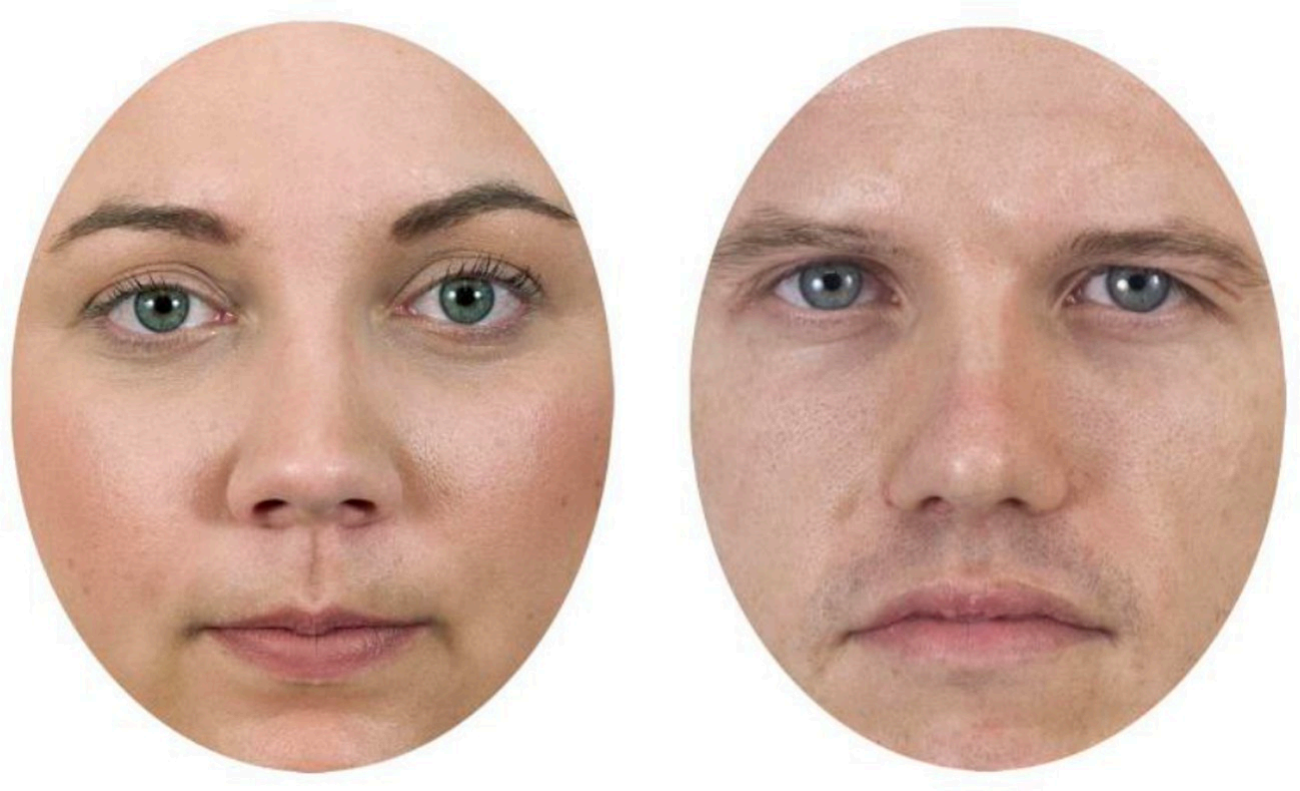
Examples of a female (left) and male (right) face used to produce the stimuli in the complex task (both setups). Note. Faces obtained from Chicago Face Database [41].

Stimuli used in the Complex Task were identical for the unimanual and bimanual setups. Pictures of 45 female and 45 male faces (taken from the Chicago Face Database, [41]) were combined into three sets of face pairs: 1) 15 image pairs consisting of two female faces, 2) 15 pairs consisting of two male faces, and 3) 15 pairs consisting of one female and one male face. The faces were pretested to ensure that female and male faces were categorised according to their respective gender category equally rapidly, and that comparable pictures were assigned to the matched and mixed gender categories. Further information regarding this process (image selection-from-database criteria, image post-processing, and image pairing procedure) is described in the project protocol (https://dataverse.no/dataset.xhtml?persistentId=doi:10.18710/WWPPCA#). The dimensions for each face pair stimulus was 300 x 508 pixels (8.5 x 14.3cm). Examples of the faces used to produce the stimuli are presented in Fig 4. The faces used were identical across both setups but were aligned with the spatial arrangement of Set 1 and Set 2 in the button task. As such, mixed face pairs in Set 1 had the female face on the left side, while mixed face pairs in Set 2 had the male face on the left side.

### 2.4. Procedure

The study was conducted at the Norwegian University of Science and Technology, Trondheim. Participants gave written informed consent prior to participation. The full experiment was composed of five blocks, but in this study we examine only the first (simple task, 45 trials [15 per stimulus category]), second (complex task 45 trials [15 per stimulus category]), and fifth (repetition of simple task 30 trials [10 per stimulus category]) block. The full procedure, which was identical for the unimanual and the bimanual setups, is described in the project protocol (https://dataverse.no/dataset.xhtml?persistentId=doi:10.18710/WWPPCA#). In all three blocks, each trial began with a gaze-contingent fixation cross being displayed in the centre of the screen. When the participant had fixated on the cross for 500ms, an image stimulus specific to the task appeared centrally on the screen and remained until a response was made. The centralisation of both stimuli and response box was intended to heighten the spatial-anatomical relationship between stimulus and response options. This centralisation is especially important for the unimanual setup, as it makes it equally motorically difficult for both left- and right-handed participants to respond using their dominant hand. Throughout the study participants were instructed to respond as quickly and accurately as possible. For the unimanual setup, responses were recorded at the point where the participant had fully pressed a single button. For the bimanual setup, responses were recorded at the point where participants had pressed the first of two buttons, with the program also noting when the second button was pressed. While analyses presented here are based on the first button press, an examination of response times showed no significant differences in using the first or second button press as the dependent measure.

Half of the participants undertook the study with the stimuli and response setup shown in Fig 3i[a] (unimanual setup) and 3ii[a] (bimanual setup), while the other half undertook the study with the inverted stimuli and response setup (Fig 3i[b] and 3ii[b]). For the Complex Task, the arrangement of the mixed face pairs was kept congruent with the spatial arrangement of the response categories.

### 2.5. Data preparation and analysis

The data preparation and analysis are briefly summarized here; more detailed information is provided in the project protocol (https://dataverse.no/dataset.xhtml?persistentId=doi:10.18710/WWPPCA#) and in S2 Appendix. Prior to analysis, both item-by-participant and by-participant data screening were used. This led to the removal of 2.49% of the data, and the exclusion of two participants’ results from the bimanual setup.

In the analyses related to the complex task all participants were included. However, due to the low number of left-handed participants, analyses related to the Simple Task were restricted to right-handed participants only to better control for motoric aspects relating to how responses were given. This means the final data set for the unimanual setup was 34 for the Complex Task (18 male; mean age 23.3 years, *SD* = 1.8) and 32 for the Simple Task (16 male; mean age 23.4 years, *SD* = 1.9), while the final data set for the bimanual setup was 30 for the Complex Task (15 male; mean age 23.9 years, *SD* = 3.6) and 25 for the Simple Task (13 male; mean age 23.0 years, *SD* = 2.0).

Statistical analyses were conducted using generalised linear mixed effects modelling in R Software (version 4.1.2) using the *glmer* function of the *lme4* package [42]. This is a strongly robust analytical approach that does not require normality, making it ideal for examinations of error rate (as it is a binomial factor; e.g., [43]) and response time data (e.g., [44]). The dependent variables examined in these analyses are Error Rate (ER, all trials), and Response Time (RT, specifically for *correct* responses). The independent variables (outlined in S2 Appendix) differed between Simple and Complex Task, and were subject to model fitting. For the analyses of the Complex Task, the mean ER and RT from the Simple Task (per response condition, averaged across both blocks) were tested as potential control variables for the matching complex task analyses (i.e., Mean ER for the ER analysis, Mean RT for the RT analysis) as both a fixed factor and as a by-participant random slope. This is in keeping with previous research (e.g., [25]), and provides a measure of cognitive cost when motoric cost is accounted for. Further, even though left-handed participants’ motoric responses were not included in the analyses of the Simple Task, they were still calculated and used on a per-participant basis as a control variable for the Complex Task. Three sets of analyses were conducted: the results of the Simple Task were analysed both separately and across setups, then finally the results of the Complex Task were examined across setups.

## 3. Results

### 3.1. Simple task

#### 3.1.1. Unimanual setup

*3*.*1*.*1*.*1*. *Error rate*. Variation in the model was so minor that no fixed effect was found to improve the model (*M*_ER_ = 0.86%; *SD* = 9.21%).

*3*.*1*.*1*.*2*. *Response time*. The model of best fit (conditional *R*^2^ = .26, AIC = 26500) contained fixed effects of Finger and Block, as well as their interaction, and had a random structure composed of random intercepts of Image, Participant, and Trial Number. The results indicated small main effects of Finger, Wald *X²* (*df* = 2) = 52.45, *p* < .001, $\eta_{p}^{2}$ *=* .03, and Block, Wald *X²* (*df* = 1) = 39.73, *p* < .001, $\eta_{p}^{2}$ *=* .02, as well as a very small two-way interaction between Finger and Block, Wald *X²* (*df* = 2) = 8.04, *p* = .018, $\eta_{p}^{2}$ *<* .01.

The main effect of Finger indicated that participants tended to respond slower with their middle finger (*M*_RT_ = 456ms, 95%CI [408ms, 508ms]) than with either their ring finger (*M*_RT_ = 423ms, 95%CI [379ms, 472ms]; *M*_*DIFF*_ **=** 33ms, 95%CI [-64ms, 129ms]) or their index finger (*M*_RT_ = 417ms, 95%CI [374ms, 465ms]; *M*_*DIFF*_ **=** 39ms, 95%CI [-57ms, 130ms]).

The main effect of Block indicated that participants tended to respond faster during the second button task (*M*_RT_ = 418ms, 95%CI [375ms, 466ms]) than during the first button task (*M*_RT_ = 440ms, 95%CI [395ms, 491ms]; *M*_*DIFF*_ **=** 22ms, 95%CI [-71ms, 116ms]), with the interaction between Finger and Block indicating that this difference was more pronounced for the middle (*M*_*DIFF*_ **=** 36ms, 95%CI[-64ms, 136ms]) than for the index (*M*_*DIFF*_ **=** 22ms, 95%CI [-71ms, 113ms]) and the ring finger (*M*_*DIFF*_ **=** 11ms, 95%CI [-79ms, 103ms]).

#### 3.1.2. Bimanual setup

*3*.*1*.*2*.*1*. *Error rate*. The model of best fit (conditional *R*^2^ > .99, AIC = 63) contained a fixed effect of Block, with a random structure composed of a random intercept of Participant. The results indicated no significant effect of Block, Wald *X²* (*df* = 1) = 0.03, *p* = .853, $\eta_{p}^{2}$ < .01. Descriptively, error rate was very low (*M*_ER_ = 0.39%; *SD* = 6.22%).

*3*.*1*.*2*.*2*. *Response time*. The model of best fit (conditional *R*^2^ = .28, AIC = 20819) contained fixed effects of Hand and Block, as well as their interaction, and had a random structure composed of a random intercept of Participant and Trial Number. The results indicated small significant main effects of Hand, Wald *X²* (*df* = 2) = 114.03, *p* < .001, $\eta_{p}^{2}$ *=* .06, and Block, Wald *X²* (*df* = 1) = 59.74, *p* < .001, $\eta_{p}^{2}$ *=* .03.

The main effect of Hand indicated that participants tended to respond slower when they were responding with both hands (*M*_RT_ = 526ms, 95%CI [471ms, 587ms]) than when responding with just their left (*M*_RT_ = 487ms, 95%CI [436ms, 543ms]; *M*_*DIFF*_ **=** 39ms, 95%CI [-72ms, 151ms]) or right hand (*M*_RT_ = 476ms, 95%CI [426ms, 531ms]; *M*_*DIFF*_ **=** 50ms, 95%CI [-60ms, 161ms]).

The main effect of Block indicated that participants tended to respond faster during the second button task (*M*_RT_ = 476ms, 95%CI [426ms, 531ms]) than during the first button task (*M*_RT_ = 509ms, 95%CI [456ms, 568ms]; *M*_*DIFF*_ **=** 33ms, 95%CI [-75ms, 142ms]).

#### 3.1.3. Comparative analyses

*3*.*1*.*3*.*1*. *Error rate*. The model of best fit (conditional *R*^2^ = .43, AIC = 299) contained fixed effect of Motoric Response and Setup, as well as their interaction, with a random structure composed of a random intercept of Participant. Overall, error rates were very low. The results indicated a small significant effect of Motoric Response, Wald *X²* (*df =* 2) = 8.92, *p* = .012, $\eta_{p}^{2}$ = .05 that was qualified by a small significant two-way interaction between Motoric Response and Setup, Wald *X²* (*df =* 2) = 6.46, *p* = .040, $\eta_{p}^{2}$ = .02.

The Motoric Response by Setup interaction indicated that participants responding unimanually produced significantly more errors with their middle finger (*M*_ER_ = 1.30%, 95%CI[0.60%, 2.82%]) compared to their index finger (*M*_ER_ = 0.07%, 95%CI[0.01%, 0.58%]; *M*_*DIFF*_ **=** 1.23%, 95%CI[0.02%, 2.81%]), and tended to produce more errors for their middle compared to their ring finger (*M*_ER_ = 0.15%, 95%CI[0.03%, 0.67%]; *M*_*DIFF*_ **=** 1.15%, 95%CI[-0.07%, 2.79%]), while participants responding bimanually produced roughly equivalent error rates for all response options (left hand *M*_ER_ = 0.16%, 95%CI[0.03%, 0.85%]; right hand *M*_ER_ = 0.11%, 95%CI[0.02%, 0.69%]; both hands *M*_ER_ = 0.11%, 95%CI[0.02%, 0.71%]).

*3*.*1*.*3*.*2*. *Response time*. The model of best fit (conditional *R*^2^ = .31, AIC = 47257) contained fixed effects of Motoric Response, Block, and Setup, as well as their interactions, with a random structure composed of random effects of Participant and Trial Number. The results indicated small main effects of Setup, Wald *X²* (*df* = 1) = 8.36, *p* = .003, $\eta_{p}^{2}$ = .02, Motoric Response, Wald *X²* (*df* = 2) = 209.48, *p* < .001, $\eta_{p}^{2}$ = .05 and Block, Wald *X²* (*df* = 1) = 92.31, *p* < .001 $\eta_{p}^{2}$ = .02. The results also indicated a very small two-way interaction between Setup and Motoric Response, Wald *X²* (*df* = 2) = 7.25, *p* = .027, $\eta_{p}^{2}$ < .01, which was qualified by a very small three-way interaction between Setup, Motoric Response, and Block, Wald *X²* (*df* = 2) = 10.68, *p =* .005, $\eta_{p}^{2}$ < .01.

The main effect of Setup indicated that participants responding unimanually (*M*_RT_ = 430ms, 95%CI [389ms, 477ms]) tended to respond more quickly than those responding bimanually (*M*_RT_ = 494ms, 95%CI [444ms, 549ms]; *M*_*DIFF*_ **=** 64ms, 95%CI [-33ms, 160ms]).

The main effect of Motoric Response indicated that participants tended to respond slower when pressing the middle response option (*M*_RT_ = 483ms, 95%CI [440ms, 531ms]) than when pressing either the left response option (*M*_RT_ = 445ms, 95%CI [405ms, 489ms]; *M*_*DIFF*_ **=** 38ms, 95%CI [-49ms, 126ms]) or the right response option (*M*_RT_ = 444ms, 95%CI [404ms, 487ms]; *M*_*DIFF*_ **=** 39ms, 95%CI [-47ms, 127ms]).

The main effect of Block indicated that participants tended to respond faster during the second button task (*M*_RT_ = 441ms, 95%CI [402ms, 485ms]) compared to the first button task (*M*_RT_ = 468ms, 95%CI [426ms, 513ms]; *M*_*DIFF*_ **=** 64ms, 95%CI [-33ms, 160ms]).

**Fig 5:**
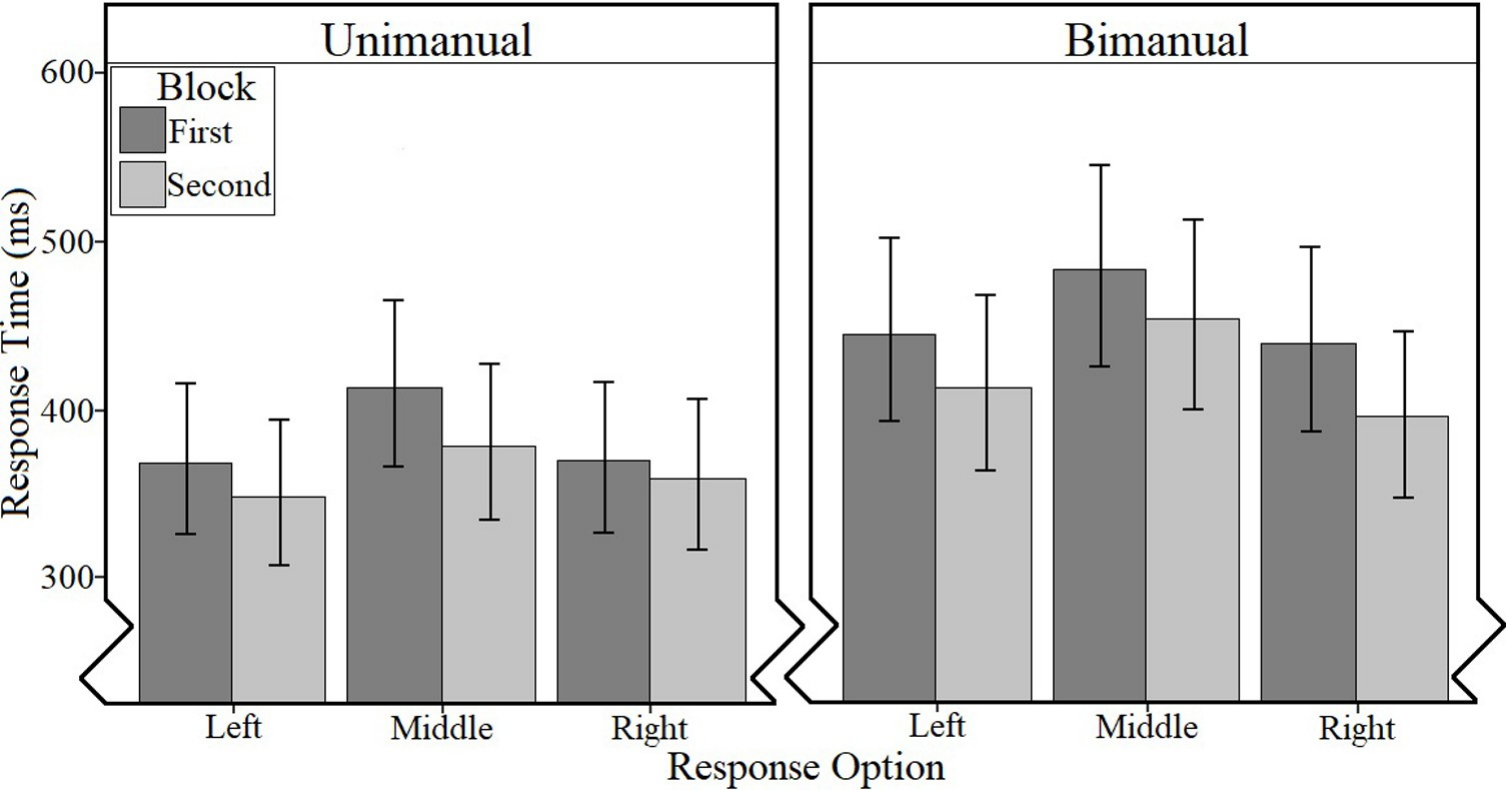
Estimated means [and 95% confidence intervals] for response time (ms) as a function of setup, Motoric Response and Block (Simple Task).

The three-way interaction between Setup, Motoric Response, and Block (Table 1 and Fig 5) indicated that the exact nature of the increase in speed between blocks differed by motoric response and setup; for the unimanual setup the largest difference between blocks was found for the middle finger (*M*_*DIFF*_ **=** 35ms, 95%CI [-59ms, 127ms]), followed by the index finger (*M*_*DIFF*_ **=** 20ms, 95%CI [-66ms, 105ms]), and smallest for the ring finger (*M*_*DIFF*_ **=** 10ms, 95%CI [-77ms, 97ms]), and for the bimanual setup the largest difference between blocks was found for the right hand (*M*_*DIFF*_ **=** 43ms, 95%CI [-58ms, 146ms]), followed by the left hand (*M*_*DIFF*_ **=** 30ms, 95%CI [-74ms, 135ms]), and smallest for the bimanual response (*M*_*DIFF*_ **=** 29ms, 95%CI [-83ms, 142ms]).

**Table 1:** Estimated means [and 95% confidence intervals] for response time (ms) as a function of setup, Motoric Response and Block (Simple Task).

		Setup	
Motoric Response	Block	Unimanual	Bimanual
Left	First	425 [383, 470]	497[447, 554]
	Second	405 [365, 449]	467 [419, 521]
Middle	First	469 [423, 519]	535 [481, 596]
	Second	434 [392, 482]	506 [454, 564]
Right	First	426 [384, 472]	492 [442, 548]
	Second	416 [375, 461]	449 [402, 500]

### 3.2. Complex task

#### 3.2.1. Error rate

The model of best fit (Conditional R² = .23, AIC = 1017) contained fixed effects of Setup, Response Condition, and Mean Simple Task Error Rate (logarithmically transformed), with the random structure composed of random intercepts of Image and Participant. The results indicated small significant effects of Response Condition, Wald *X²* (*df =* 2) = 19.79, *p <* .001, $\eta_{p}^{2}$ = .03, and Setup, Wald *X²* (*df =* 1) = 8.90, *p =* .001*, $\eta_{p}^{2}$* = .01.

The main effect of Response Condition indicated that participants produced significantly more errors when responding to mixed face pairs (*M*_ER_ = 5.99%, 95%CI [4.18%, 8.51%]) than when responding to either male (1.91%, 95%CI [1.15%, 3.14%]; *M*_*DIFF*_ = 4.08%, 95%CI [1.04%, 7.36%]) or female (2.52%, 95%CI [1.58%, 4.01%]; *M*_*DIFF*_ = 3.47%, 95%CI [0.17%, 6.93%]) face pairs, and tended to produce more errors when responding to female compared to male face pairs (*M*_*DIFF*_ = 0.61%, 95%CI [-1.56%, 2.86%]).

The main effect of Setup indicated that participants responding unimanually (*M*_ER_ = 4.25%, 95%CI [3.02%, 5.94%]) tended to produce more errors than those responding bimanually (*M*_ER_ = 2.11%, 95%CI [1.36%, 3.24%]; *M*_*DIFF*_ = 2.14%, 95%CI [-0.22%, 4.58%]).

#### 3.2.2. Response time

The model of best fit (Conditional R² = .33, AIC = 37186) contained fixed effects of Setup, Response Condition, and Mean Simple Task RT (logarithmically transformed), as well as an interaction between Setup and Response Condition, with the random structure composed of random intercepts of Image, Trial Number, and Gender, and a random slope of Mean Simple Task RT by Participant (logarithmically transformed). Mean Simple Task RT had no significant effect, Wald *X²* (*df* = 1) = 3.74, *p* = .053, $\eta_{p}^{2}$ = .01. The results indicated small significant effects of Response Condition, Wald *X*² (*df* = 2) = 30.06, *p* < .001, $\eta_{p}^{2}$ = .03, and Setup, Wald *X*² (*df* = 1) = 5.97, *p* = .015, $\eta_{p}^{2}$ = .01, as well as a small two-way interaction between Setup and Response Condition, Wald *X*² (*df* = 2) = 14.31, *p* < .001, $\eta_{p}^{2}$ = .01.

The main effect of Response Condition indicated that participants tended to respond slower to mixed face pairs (*M*_RT_ = 1089ms, 95%CI[931ms, 1274ms]) than to female (*M*_RT_ = 939ms, 95%CI [802ms, 1100ms]; *M*_*DIFF*_ = 150ms, 95%CI [-169ms, 472ms]) or male faces (*M*_RT_ = 923ms, 95%CI [788ms, 1080ms]; *M*_*DIFF*_ = -149ms, 486ms]).

**Fig 6:**
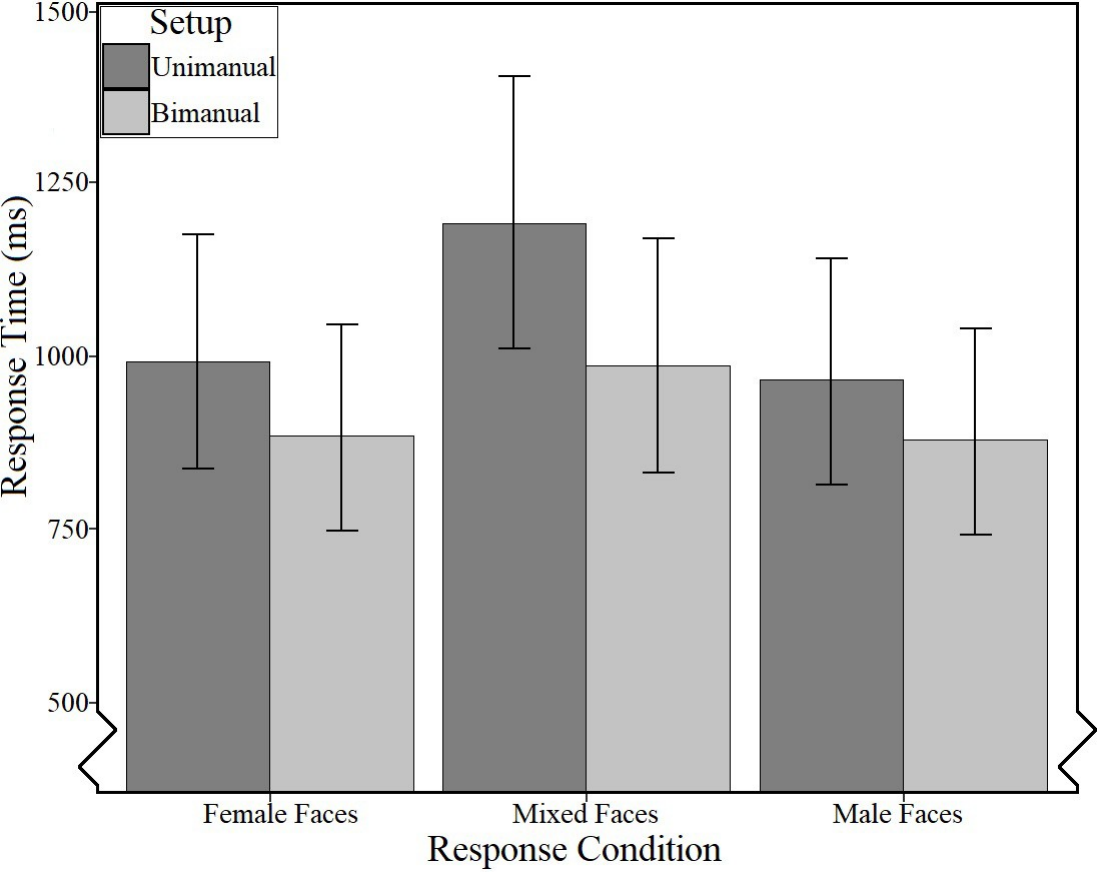
Estimated means [and 95% confidence intervals] for response time (ms) as a function of setup and response condition (complex task).

The main effect of Setup indicated that participants responding unimanually (*M*_RT_ = 1041ms, 95%CI [884ms, 1226ms]) tended to respond more slowly than those responding bimanually (*M*_RT_ = 914ms, 95%CI [556ms, 1076ms]; *M*_*DIFF*_ = 127ms, 95%CI [-192ms, 670ms]), with the Setup by Response Condition interaction (Table 2 and Fig 6) indicating that this difference was greatest for the mixed gender faces (*M*_*DIFF*_ = 205ms, 95%CI [-159ms, 573ms]), followed by the female faces (*M*_*DIFF*_ = 106ms, 95%CI [-210ms, 428ms]), and was smallest for the male faces (*M*_*DIFF*_ = 85ms, 95%CI [-255ms, 399ms]).

**Table 2:** Estimated means [and 95% confidence intervals] for response time (ms) as a function of setup and response condition (complex task).

	Setup	
Response Condition	Unimanual	Bimanual
Female	991 [836, 1176]	885 [748, 1046]
Mixed	1191 [1010, 1405]	986 [832, 1169]
Male	964 [814, 1142]	879 [743, 1039]

## 4. Discussion

The aim of this study was to investigate the comparative adequacy of two alternative response layouts for use with three-alternative CRT tasks, where one stimulus category presents a combination of the other two stimulus categories. Both a reference button task (Simple Task) and a face pair task (Complex Task) were examined. The reference button task provided a measure of motoric difficulty, which was then used as a covariate in examining the adequacy of the S-R setups for use in the face pair task.

The results can be summarized as follows: (1) during the reference button task, participants responded slower with their middle finger (in line with H1) or bimanually (in line with H2) than with the other respective responses (digit/ring finger; left/right unimanually). Further, error rate was very low for both setups. (2) In line with H3, the comparison analysis between the setups for the reference button task indicated that participants using the unimanual setup responded significantly faster than those using the bimanual setup, pointing to lower motoric costs when responding with one hand. This indicates that, for the reference button task, adequacy relating to response time was better for the unimanual setup. Mirroring the results for the per setup analyses, middle responses were slower than left or right responses, but there was an unexpected three-way interaction effect indicating diverse task repetition effects by setup. Further, in relation to error rate, the comparative results indicated considerably higher error rates for the middle finger specifically than for any other response option across both setups. (3) Even when controlling for differences in motoric costs, error rates and response times in the face pair task were higher for the mixed compared to the matched stimuli, indicating—in line with H4—that categorising overlapping stimulus conditions is more difficult than categorising independent stimulus conditions. This in turn supports the notion that responding to mixed stimuli is more challenging for encoding and/or decision making processes. (4) When controlling for differences in motoric costs, error rates and response times in the face pair task were higher for the unimanual setup overall compared to the bimanual setup (in line with H5). This indicates that adequacy relating to error rate and response time for this task was better for the bimanual setup. Taken together, these results suggest that, when a two-dimensional conceptualisation of stimulus categories is possible, a bimanual setup can be more appropriate to use.

The three-way interaction in the analysis of the response times for the reference button task indicates different task repetition effects (i.e., changes in response time between the first and second reference button blocks) for the different setups. For the unimanual setup, repeating the task reduced the response time to a higher degree for the finger with the slowest (middle finger) and the finger with the fastest (digit finger) motoric response than for the ring finger. This is unexpected as repetition effects could have been expected for the ring finger as well, and suggests that the outer finger advantage [33, 35] reduced motoric costs associated with the ring finger near to the biological baseline already during the first button task block. For the bimanual setup, repeating the task reduced the response times to a slightly higher degree for the right response than for the left and the bimanual response. This indicates that (the motorically more costly) bimanual synchronization and the (for the majority of participants non-dominant) left hand response has less potential for improvement in the same timeframe—that is, it would take more repetitions to improve bimanual and left-hand responses to the same level as right-hand responses.

Across the analyses, middle responses were slower (and often less accurate) than outer responses. We expected such findings based on finger motoric costs, the “outer finger”-effect [33, 35], the costs associated with synchronisation of two hands in the reference button task [34], and due to matched vs. mixed categories effects in the face pair task. Concerning the reference button task, another explanation for this effect is that the matched stimuli produced a processing advantage, as they contain peripheral visual cues that may provide more salient spatial information than the centrally presented cues in the mixed stimuli (e.g., [45]). This would mean that spatial S-R compatibility was higher for matched stimuli. If this is the case, the reference button task can be interpreted as a simple RT task with spatial cues, where lateralisation may facilitate responses to the outer buttons. With reference to either task, an alternative (or additional) explanation for the higher processing costs associated with middle/mixed stimuli is the spatial coding of response options (e.g., [46, 47]). As the outer/matched stimuli elicit responses completely corresponding to a spatially located hand or finger, participants may have spatially associated the outer/matched stimuli with a specific ‘side’, whereas with the middle/mixed stimuli they had to overcome this spatial coding bias by responding with a central finger (unimanual setup) or bimanually (bimanual setup). This poses a challenge for tasks that include overlapping categories, i.e. where responses cannot be counterbalanced across conditions and hence other solutions need to be in place to take this imbalance into account.

In the face pair task, the difference in response time between the matched and the mixed face pairs was more pronounced for the unimanual than in the bimanual setup. As research suggests that S-R compatibility is highest when mental representations and motor outputs most closely match (e.g., [9, 48]), this unexpected finding lends further support to the notion that our face pair task is designed in a way that is more adequately represented in a two-dimensional structure in which the bimanual response is a motoric analogue to the overlapping stimulus category (mixed face pairs).

In conclusion, this research adds to the literature by examining S-R compatibility for response setups used with purposefully ‘overlapping’ stimulus categories. While both response setups have the challenge of the middle response, overall the unimanual setup had a higher adequacy than the bimanual setup for the motoric reference button task, while the bimanual setup had a higher adequacy than the unimanual setup for the face pair task. In keeping with previous research, (e.g., [18]), these results demonstrate the importance of a careful consideration of the compatibility between the stimulus and response set, by showing that response setups with higher S-R compatibility facilitate the selection of response categories to the degree that they can more than compensate for the accompanying increased motoric cost. In other words, the use of a more complex setup that, due to better reflecting the manner in which participants conceptualise response categories, has high S-R compatibility may be preferred to the use of a motorically easier setup with lower S-R compatibility. This seems especially true for the more demanding ‘overlapping’ category, with our results suggesting that its complexity is better contained when some response alternatives are allocated to one hand and other alternatives to the other hand.

Our specific focus has been on deciding on a setup for a distinct task (face pair task) which was developed with the aim to overcome the limitations of binary response tasks when investigating the activation and processing of gender-related information. Even though that has put certain constraints on the design alternatives that were investigated, we still consider these findings relevant for the discussion of complex experimental paradigms, especially as this is highly exploratory research. As such, while this study is limited to a single paradigm, we believe that it should be generalisable to other paradigms in which a three-category approach is used, especially when one of those is an overlapping category.

## Supporting information

S1 Appendix Eye tracking data preparation and analysis. (DOCX)

S2 Appendix Data preparation—error rate and response times. (DOCX)
