# Supplementary material for: Exploring the comparative adequacy of a unimanual and a bimanual stimulus-response setup for use with three-alternative choice response time tasks
Source: PLoS One. 2023 Mar 15;18(3):e0281377. doi: 10.1371/journal.pone.0281377 (PMC10016697; doi:10.1371/journal.pone.0281377)
Supplement: S1 Appendix — (DOCX) [file pone.0281377.s001.docx]

# S1 APPENDIX A

# EYE TRACKING DATA PREPARATION AND ANALYSIS

The analyses for Visual Exploration (i.e., eye-tracking) included only those trials that were recorded during the Complex Task *and* for which the given response was correct.

## A.1. Data Preparation

Eye-tracking data was examined in two ways; firstly, Target Visits, and secondly, Relative Fixation Duration. Together, these analyses examine participants’ Visual Exploration behaviour. Fixation data was first used to assess the sequence in which the two images were explored. Three fixation locations were defined: left face, right face, or outside these regions. Since the fixation cross at the start of each trial was located outside and between the two image regions, participants would begin each trial with their gaze in a neutral position. This dataset was then subject to within-item exclusion, with fixations within each trial that were in the ‘outside’ region, were shorter than 80ms, or were longer than 800ms excluded from analysis. This accounted for 7.93% of all fixations, although (due to the nature of the analysis) not the removal of any full trials. For a detailed discussion of Visual Exploration behaviour, see the project protocol (<https://dataverse.no/dataset.xhtml?persistentId=doi:10.18710/WWPPCA>#).As with the analyses of error rate and response time, the statistical analyses for eye tracking were conducted using linear mixed effects modelling in R Software (Version 4.0.3) using the *lmer* function of version 1.1-23 of the lme4 package [1]. For analyses of Visual Exploration, the results for the two setups were modelled together. In keeping with the other analyses, initial models that capture the maximal random effects structure as justified by the design were defined. These were composed of all experimental factors (Response [FF, MM, Mixed], Spatial Arrangement [FF left vs. FF right], Participant Gender [female vs. male], and Setup [Bimanual vs. Unimanual]), as well as their two- and three-way interactions, with a random structure composed of random intercepts (Participant and Trial Number) and slopes (each fixed factor by each random factor), with model refinement following the approach outlined in the project protocol (<https://dataverse.no/dataset.xhtml?persistentId=doi:10.18710/WWPPCA>#). P-values for the best fitting models were obtained through lme4 in conjunction with the effects package [2].

## A.2. Visual Exploration of Stimuli

**A.2.1. Target Visits.** The model of best fit (Conditional R² = .23) included a fixed effect of Response, as well as random intercepts for Participant and Image, and a random slope of Response by Participant. The results indicated a large and significant main effect of Response (Wald *X*² = 33.92, *p* < .001, $\omega_{p}^{2}$ = .389); participants performed a higher number of target visits for Mixed stimuli (*M* = 2.39, 95%CI [2.28 to 2.50]) than for either FF stimuli (*M* = 2.12, 95% CI [2.02 to 2.21]; *M*_Diff_ = 0.27, 95%CI [0.07 to 0.48) or MM stimuli (*M* = 2.13, 95% CI [2.03 to 2.23]; *M*_Diff_ = 0.26, 95%CI [0.05 to 0.47]). No significant difference in target visits was observed between FF and MM stimuli (*M*_Diff_ = 0.02, 95% CI [-0.18 to 0.21]).

**A.2.2. Total Fixation Duration.** The model of best fit (Conditional R² = .17) included fixed effects of Response, Setup, and Trial Number, as well as random intercepts of Participant and Image, and random slopes for Response and Trial Number by Participant. The results showed a significant medium-sized effect of Response (Wald *X*² = 33.92, *p* < .001, $\omega_{p}^{2}$ = .003); participants spent a larger percentage of time per trial fixating on faces for Mixed stimuli (*M* = 82%, 95%CI [80% to 84%]) than for either FF stimuli (*M* = 80%, 95%CI [78% to 82%]; *M*_Diff_ = 2%, 95%CI [-2% to 7%]) or MM stimuli (*M* = 79%, 95%CI [77% to 82%]; *M*_Diff_ = 3%, 95%CI [-2% to 7%]). No significant difference was observed between FF and MM stimuli (*M*_Diff_ = 0%, 95%CI [-4% to 5%]).

## A.3. Discussion of Results

These results support the finding that the mixed stimuli were more cognitively demanding than the matched stimuli. Participants had more frequent, and longer, target region visits for mixed compared to matched face pairs in both response layouts, meaning that mixed face pairs were more actively explored. These results indicate systematically higher processing costs associated with responding to mixed stimuli. Further, as Setup was not significant for the results of both Target Visits and Total Fixation Duration, this indicates that participants used the same visual exploration strategy in both setups. The increases in visits and duration explains the increase in response times for mixed stimuli compared to matched stimuli in both setups, indicating that facial scanning was an active part of the cognitive translation process.

## References

1. Bates D, Mächler M, Bolker B, Walker S. Fitting Linear Mixed-Effects Models Using lme4. J Stat Softw. 2015;67. doi:10.18637/jss.v067.i01

2. Fox J, Weisberg S. An R companion to applied regression. Third edition. Los Angeles: SAGE; 2019.
